# Evaluation of Curve Flexibility Using Scoliotic Spines Generated From a Generic Spine Model

**DOI:** 10.1002/cnm.70213

**Published:** 2026-09-15

**Authors:** Yifan Su, Athanasios I. Tsirikos, Vasileios Koutsos, Pankaj Pankaj

**Affiliations:** ^1^ Institute for Bioengineering, School of Engineering, The University of Edinburgh Edinburgh UK; ^2^ Scottish National Spine Deformity Centre – Royal Hospital for Children and Young People Edinburgh UK; ^3^ Institute for Materials and Processes, School of Engineering, The University of Edinburgh Edinburgh UK

**Keywords:** adolescent idiopathic scoliosis, finite element analysis, scoliosis models, spine biomechanics, spine flexibility

## Abstract

Curve flexibility is critical for preoperative planning in adolescent idiopathic scoliosis (AIS) as it guides surgical strategy and selection of appropriate release manoeuvres. Although finite element (FE) modelling has been widely used to investigate spinal biomechanics, its application to spinal flexibility assessment remains underexplored. This study developed AIS FE models to evaluate the effects of sequential spinal release techniques on curve flexibility under fulcrum bending radiographs (FBR) and supine traction radiographs (STR). A validated generic adolescent spine model was deformed using a displacement‐based shaping approach to generate three representative AIS spines. Curve flexibility was assessed computationally under simulated FBR and STR conditions. Five sequential surgical release manoeuvres were then simulated. To estimate the required traction force for STR, three cases of AIS patients were analysed to determine the traction force that reproduced the clinically reported flexibility. Three generic‐derived scoliotic spines and three patient‐based curves were successfully generated. The effective STR traction force was estimated as 376.6 N. For curve flexibility assessments, disc release produced the largest flexibility gain, while facetectomy produced the least improvement. Under STR, flexibility increased progressively with stepwise releases. Under FBR, flexibility responses were more curve‐type dependent. This study provides a computational approach for developing AIS models, assessing AIS curve flexibility and comparing spinal release strategies. Generic‐derived scoliosis models enabled controlled curve flexibility comparisons across various curve types and surgical manoeuvres; thus, they may assist in eliciting answers to common clinical questions and support preoperative planning.

## Graphical abstract

This study presents a modelling procedure to evaluate curve flexibility in adolescent idiopathic scoliosis. Models were generated from a previously assessed adolescent spine model, with patient radiographs used to estimate traction forces. Sequential spinal release simulations were conducted. The framework enables controlled comparison of flexibility across curve types and release strategies.

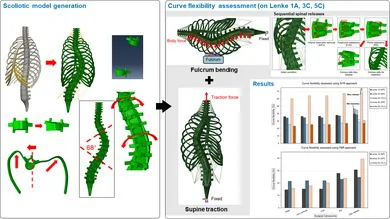

## 1 Introduction

Adolescent idiopathic scoliosis (AIS) refers to a three‐dimensional spine deformity of unknown aetiology that develops during adolescence and is characterised by abnormal spinal curvatures associated with axial vertebral rotations [1, 2]. Curve flexibility, the degree to which a scoliotic curve can be reduced under passive or assisted manoeuvres [3], plays an essential role in treatment planning. Based on the radiographic behaviour, curve flexibility reflects the structural stiffness of the spine, which governs what correction is realistically achievable, thus, it influences implant strategies, resulting in varied spinal releases or osteotomies in AIS surgery [3, 4, 5].

Multiple radiographic techniques are used to estimate curve flexibility, each involving different constraints and forces on the patient's spine. Fulcrum bending radiography (FBR) concentrates corrective bending at the scoliotic curve by providing an external fulcrum under apical levels of scoliotic spines [6, 7]. FBR often reveals greater correction of thoracic curves than standard side‐bending radiography (SBR) [7, 8, 9] since FBR solely relies on the patient's body weight, while SBR asks patients to bend themselves, which can be influenced by personal pain tolerance. By contrast, supine traction radiography (STR) applies more global, longitudinal forces along the spine and may better reflect the curve flexibility [10, 11]. The STR approach is often performed with the patient lying supine while longitudinal traction is applied to the spine. In AIS, this approach is often more applicable for young patients compared to FBR and SBR as they are more likely to have difficulty in executing voluntary side‐bending [12]. However, the traction force applied is not usually standardised in clinical practice and is frequently determined by the surgeon's maximum effort.

As reported, curve flexibility of scoliosis varies widely depending on the radiographic techniques used, as well as patient soft tissue behaviour, curve patterns and severities [6, 13, 14]. This variability makes direct comparisons across biomechanical studies on flexibility challenging and limits the understanding of the effectiveness of surgical techniques in improving flexibility.

While clinical imaging provides essential patient‐specific estimates, it cannot directly reflect the mechanical contributions of specific components to curve flexibility (e.g., spinal joints, soft tissues, ligaments, ribcage). It is, therefore, insufficient to test the effectiveness of surgical strategies (e.g., selective releases of the spine, osteotomy, implant conducts). Computational modelling offers a powerful method to evaluate the biomechanics of these surgical techniques. With appropriate models and target boundary conditions applied, finite element (FE) modelling allows for systematically varying osteotomies involving facet joints or vertebral posterior columns, as well as resections of intervertebral discs (IVDs) or ribs [15, 16]. The FE analysis can further quantify biomechanical variables to assess how osteotomies or spinal releases alter curve flexibility. Previous FE studies have quantified changes in spinal range of motion and load distribution following osteotomy, including the effects of different osteotomy levels on spine [17, 18]. Patient‐specific studies are particularly valuable for predicting individual curve progression during growth [19] or evaluating the effects of spinal correction and subsequent growth modulation in specific patients [20]. However, patient‐specific scoliosis models are unsuitable for answering a range of common clinical questions related to correction for typical AIS patterns.

Thus, this study aims to: (1) generate typical AIS patterns encountered in clinical practice for flexibility assessments by morphing a fully assessed generic adolescent FE spine model [21]; (2) estimate the traction forces required during STR to enable the subsequent curve flexibility assessments; (3) evaluate the curve flexibility change due to different spinal release techniques under two radiographic techniques (FBR and STR), so as to reflect the mechanical contributions of different spinal components to curve flexibility, thereby contributing to decision making and optimisation of surgical correction strategies for AIS treatment.

## 2 Methods

### 2.1 Scoliosis Model Generation

**FIGURE 1:**
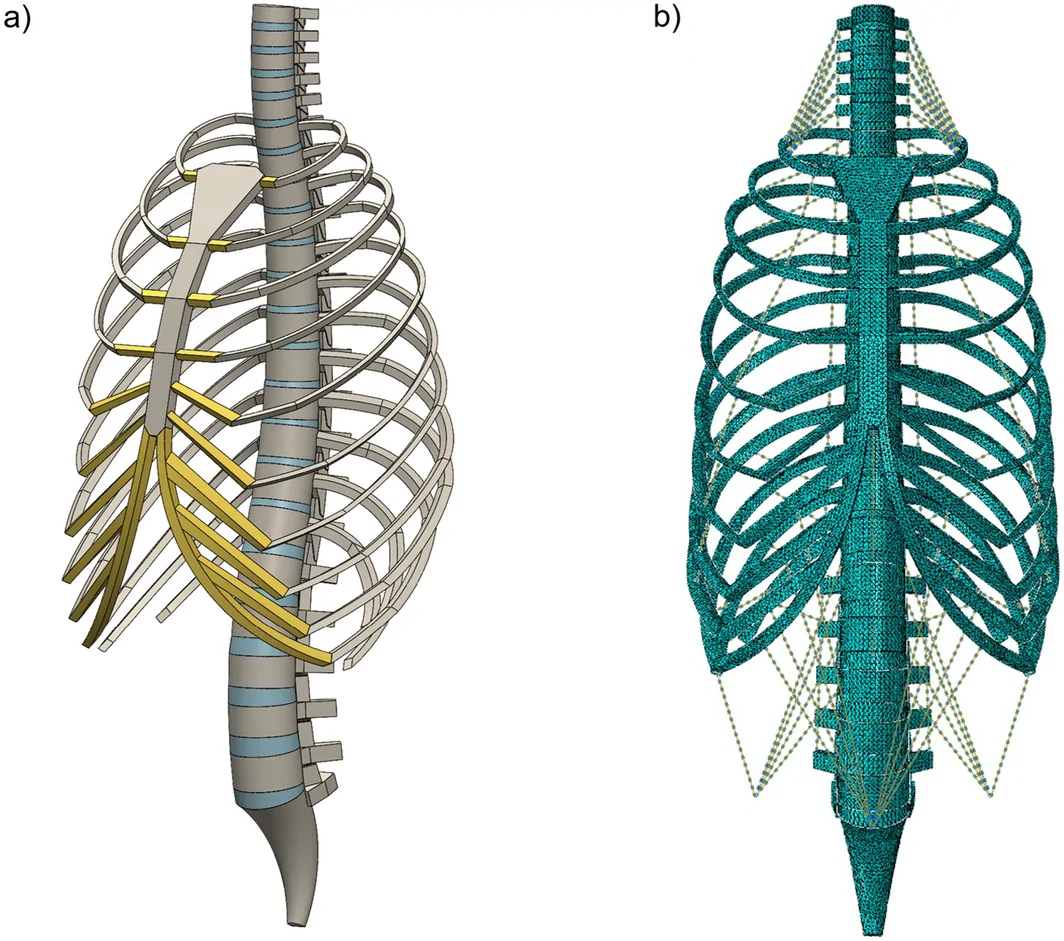
A novel generic adolescent FE spine model. (a) Parametric design of the adolescent spine, involving C3—L5 spinal column with ribcage and sacrum. (b) The elastic component attachments (ligaments and musculature), with two imaginary pelvis points positioned for the abdominal muscle attachment.

The study employed a fully assessed generic adolescent FE spine model that included the thoracolumbar spine (T1‐L5), the lower cervical region (C3‐C7), the sacrum and the rib cage [21]. The generic model is a parametric design of a normal 15‐year‐old adolescent spine (Figure 1). The generic model includes seven types of ligaments using spring elements, and the muscles were represented using linear elastic elements to account only for their passive mechanical contribution. The model has previously been assessed under various loading conditions and shown to satisfactorily replicate the structural behaviour and realistic biomechanical responses of the spine. A summary of material properties used for developing the generic FE spine model is provided in Table S1.

**FIGURE 2:**
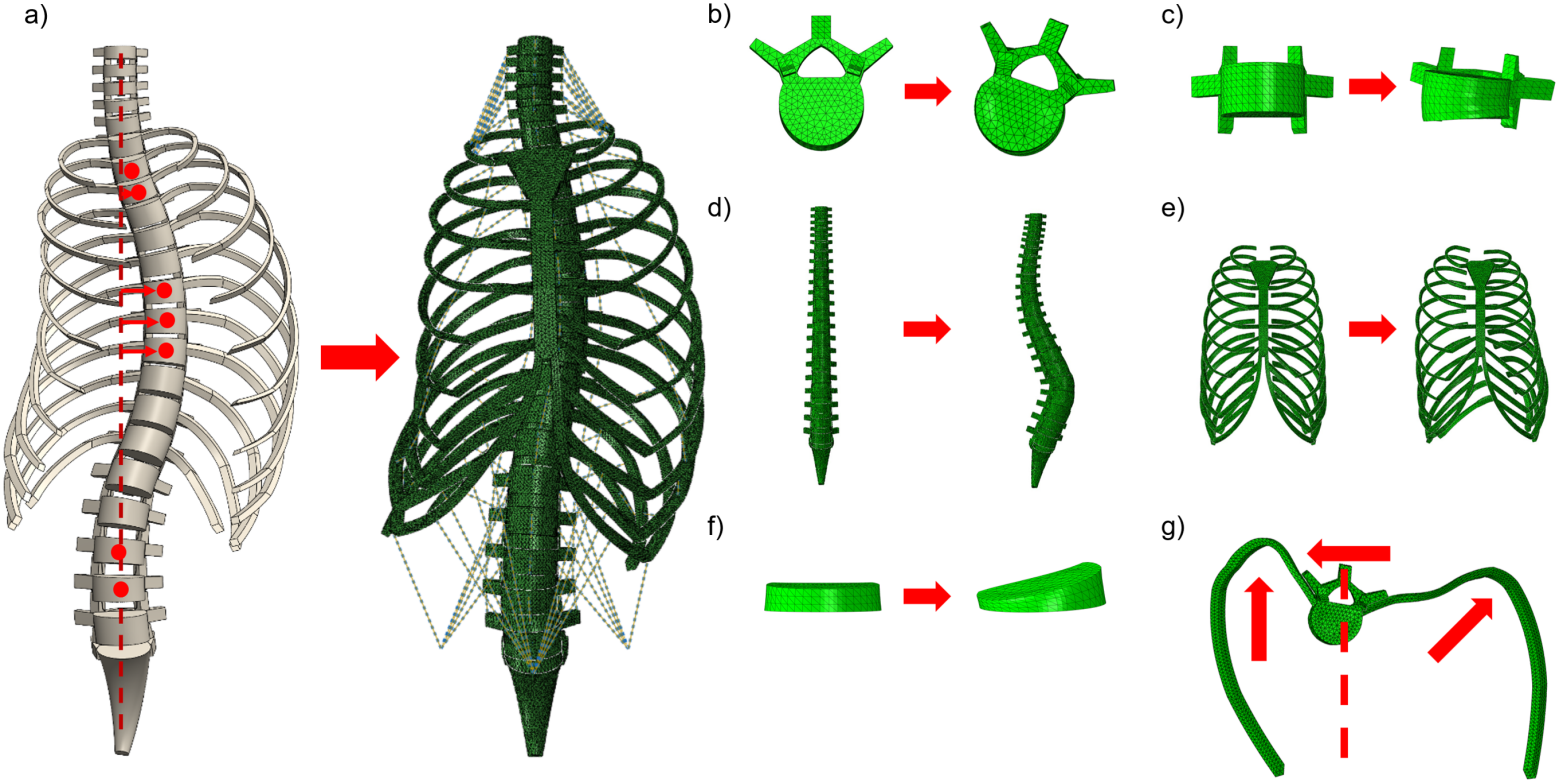
Morphological features reproduced in generated scoliosis models. (a) A prescribed scoliotic shape was used to determine the required vertebral displacements. (b) Vertebral rotation. (c) Wedged vertebra. (d) Curved spine. (e) Morphed ribcage. (f) Wedged IVD. (g) Rib hump occurred due to thoracic rotation.

This generic model was morphed to generate scoliosis models, with the aim of reproducing the morphological characteristics of scoliosis [1, 2] (Figure 2): vertebral rotation—vertebrae at apical levels were rotated towards the convex side of the curve; vertebral wedging—one or more vertebrae are compressed to become wedge‐shaped; vertebral displacements—vertebrae shift out of normal spine alignment, disrupting normal curves (kyphosis and lordosis); IVD wedging—IVDs become wedge‐shaped due to the asymmetric compression of collapsed vertebrae; ribcage deformation—ribs are displaced posteriorly and laterally due to the axial rotation of thoracic vertebrae, creating asymmetric humps.

To generate clinically representative scoliotic spines from a generic full‐spine model, and also to enable subsequent FE analyses using the generated scoliosis models, we adopted a displacement‐based shaping approach to obtain scoliotic geometries. During the shaping procedure, relatively compliant material properties were temporarily assigned to facilitate the deformation of normal spine towards the prescribed scoliotic geometry. Vertebral displacements were obtained from a prescribed scoliotic geometry (Figure 2a) and applied at apical levels to guide the coronal and sagittal deviations of the spine. In parallel, the relative stiffness between the ribcage and the spinal column was modified such that the ribcage remained globally more rigid than the spine, thereby allowing axial rotation to appear along with the spine bending. Rib deformity also emerged from the mechanical interaction between the ribcage and rotated vertebrae and curved spinal column during the morphing process, without additional prescribed constraints applied to the ribs.

Vertebral wedging is a chronic biological process of structural change [22] rather than an instantaneous geometrical response. In AIS, vertebral wedging is commonly linked to growth modulation driven by sustained asymmetric loading over time [23]. Therefore, wedging features were approximated mechanically not only through asymmetric compression of vertebral and intervertebral components during the shaping procedure, but also by temporarily modifying the relative stiffness between the vertebrae and IVDs to promote greater compressive deformation in vertebral bodies relative to the IVDs.

After the desired scoliotic deformation was obtained, the nodal coordinates of the resulting mesh were extracted and used to update the corresponding nodal coordinates of the original (normal) spine geometry by replacing the node coordinates in the *Abaqus* input file (Dassault Systèmes, USA). The temporary material modifications used during the shaping procedure were therefore not retained in the subsequent FE analyses. The updated normal baseline model adopted the generated scoliotic geometry, while retaining the normal material properties taken from the literature [21], together with the original set of ligament and muscle definitions. These scoliosis models developed from a generic normal spine model are henceforth referred to as ‘generic‐derived’ models.

Three typical scoliosis curves, Lenke Types 1A, 3C and 5C, were modelled to represent the main anatomical distributions of coronal deformity: major thoracic curve, double major curves and thoracolumbar/lumbar curve. These three types were selected to cover distinct biomechanical scenarios relevant to the flexibility assessment. Lenke 1A represents thoracic deformity, where curve flexibility is influenced by ribcage constraint. Lenke 5C represents thoracolumbar/lumbar deformity, where lumbar mobility and the absence of ribcage may produce a different response. Lenke 3C represents coupled structural thoracic and lumbar curves.

### 2.2 Flexibility Tests

**FIGURE 3:**
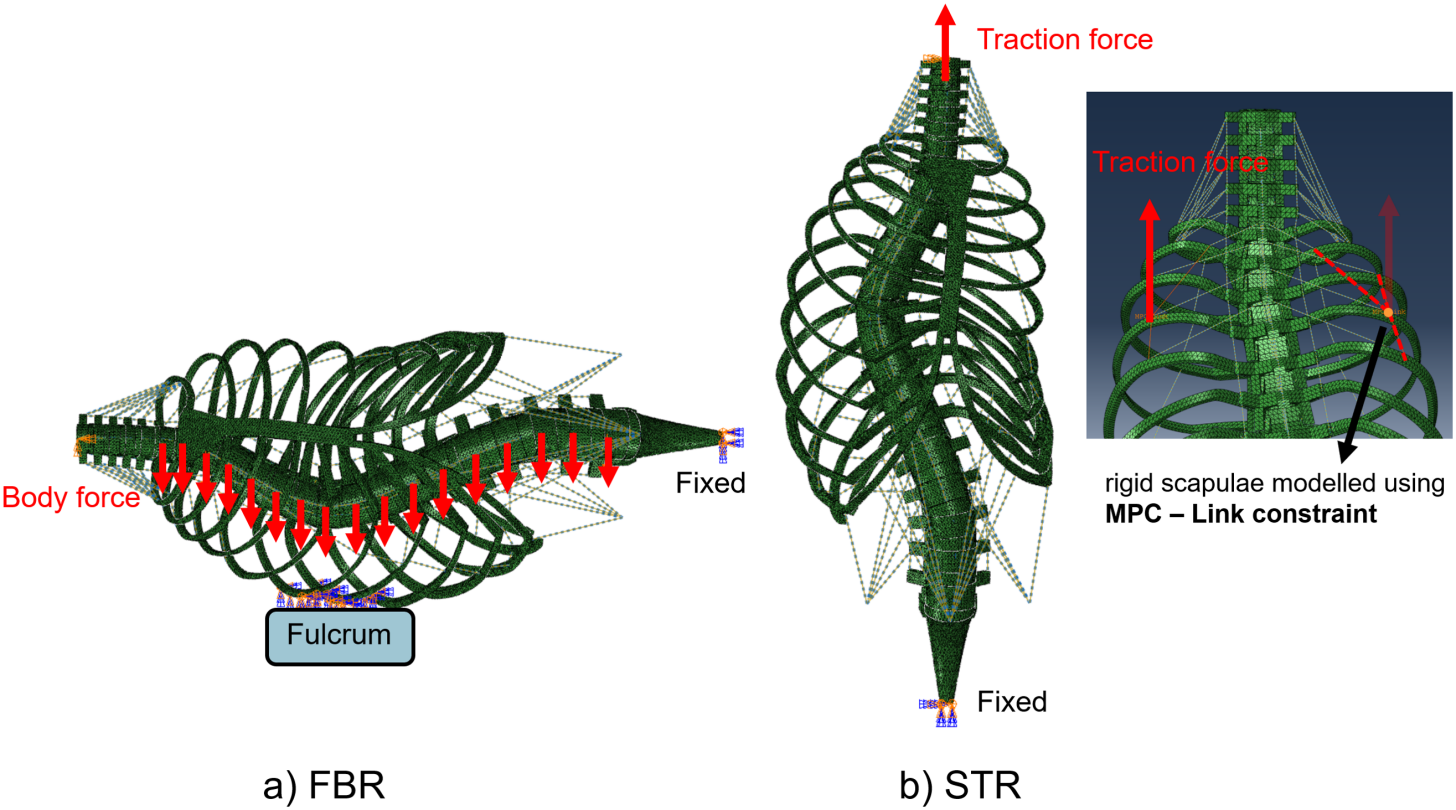
Boundary conditions for curve flexibility assessment. (a) Fulcrum bending: constraints were applied to selected ribs to represent fulcrum contact, and the bottom of the sacrum was fixed; body forces were distributed along the spinal column from T1 to L5, directing towards the fulcrum to reflect the patient lying on the support. (b) Supine traction: traction force was applied at the cervical region and from the axillae.

Two radiographic techniques were numerically simulated to assess curve flexibility (Figure 3). The first was the FBR approach (Figure 3a), while the loads applied to the spine were purely body weights. The boundary conditions imposed were constraints on the convex side of the ribcage to represent the radiographic setup, placing a fulcrum under the curve apex of the spine [6]. The 50.8% of total body weight was distributed along the spine [24], including the weight of the head and upper limbs. An example body weight of a 15‐year‐old AIS patient was employed (52.9 kg) [25]. The second radiographic method was the STR approach (Figure 3b), in which an axial traction load was applied either at the cervical region or via pulling from the axillae, consistent with common clinical practice [26, 27]. In FE models, traction was applied at the superior surface of C3 and alternatively from the rigidly modelled scapulae, which was simulated using multiple points constraint (MPC) of link component to connect T2 to T5 ribs (Figure 3b) [28]. However, the magnitude of the traction force is not standardised and is not often reported in previous studies. Therefore, the traction force required to perform STR in this study was unknown.

### 2.3 Patient‐Based Curve Generation and Traction Force Estimation

The generic‐derived models were intended to address a standardised biomechanical question by imposing different scoliosis curve patterns on the same baseline adolescent spine model (i.e., conceptually representing the same patient presenting with different curves) and evaluating how the same sequence of surgical procedures altered curve flexibility in each case.

**FIGURE 4:**
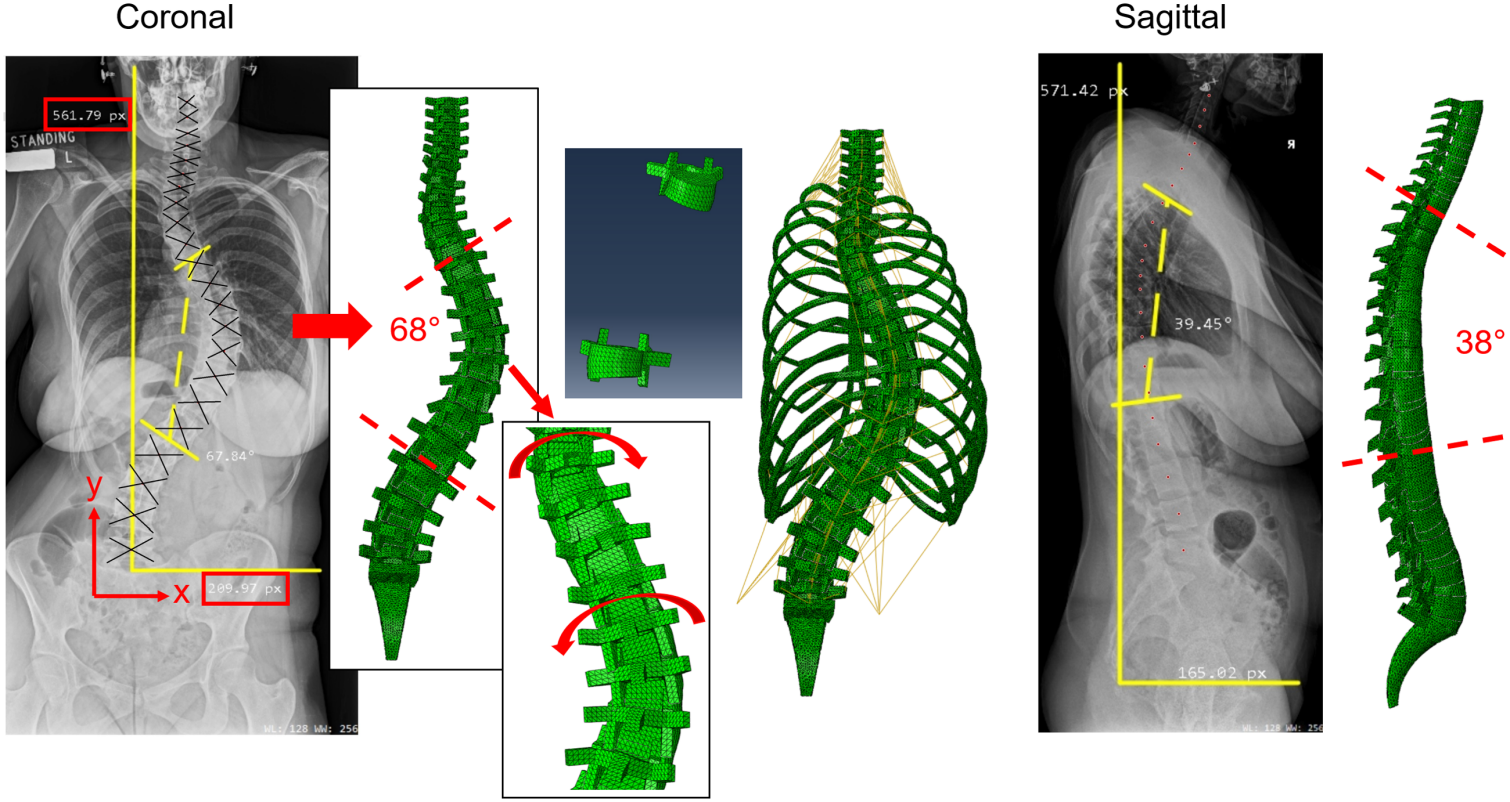
Patient‐based curve generation. Vertebral displacements were applied at each spine level in both the coronal and sagittal planes to reproduce the patient curves.

Based on the generic‐derived model generation procedure, ‘patient‐based’ (similar scoliotic pattern and not patient‐specific) models were developed. As a derivative application of the proposed shaping method, patient‐based models served as rapid approximations of the corresponding radiographic curve characteristics of selected clinical cases. This enabled a quick assessment of the associated biomechanical response, without the need for fully patient‐specific anatomical reconstruction, which would have necessitated the laborious creation of FE models from 3D scans. In this study, these patient‐based models were used only to estimate the traction force required to reproduce the clinically diagnosed STR flexibility. The patient‐based curves were generated by prescribing vertebral displacements at each spine level in both the sagittal and coronal planes (Figure 4).

To determine the traction force for the STR method, prescribed traction displacements were applied at the cervical region or axillae to obtain the corresponding reaction forces. Patient‐based curves were generated based on three clinical cases. For each case, tractions were applied to certain distances to reproduce a similar flexibility rate as reported in the corresponding clinical records. Two traction applications at the cervical region and axillae were simulated as alternative STR loading conditions and were not applied simultaneously.

The radiographs used in this study were from the historical AIS patients who had been treated and discharged from the Service and were fully anonymised. The resulting reaction forces were recorded and used to define an appropriate traction force for the following STR tests. Since only the curve features were captured, these models should not be regarded as fully patient‐specific anatomical reconstructions.

The prescribed vertebral displacements were derived by first establishing a coordinate system on the radiographs (Figure 4) and extracting the vertebral centroids, which were defined in pixel units. The spine length (50.9 cm) of the generic baseline model [21] was then scaled to match the radiographic spine length. The resulting vertebral centroid coordinates were subsequently used to calculate the prescribed vertebral displacements.

A consistent Cobb angle measuring system was employed to determine the curve flexibility changes. The two most inclined vertebrae were identified and extracted from the scoliosis model [29], and the corresponding endplate planes were defined to measure the Cobb angle.

### 2.4 Surgical Manoeuvres in Flexibility Tests

**FIGURE 5:**
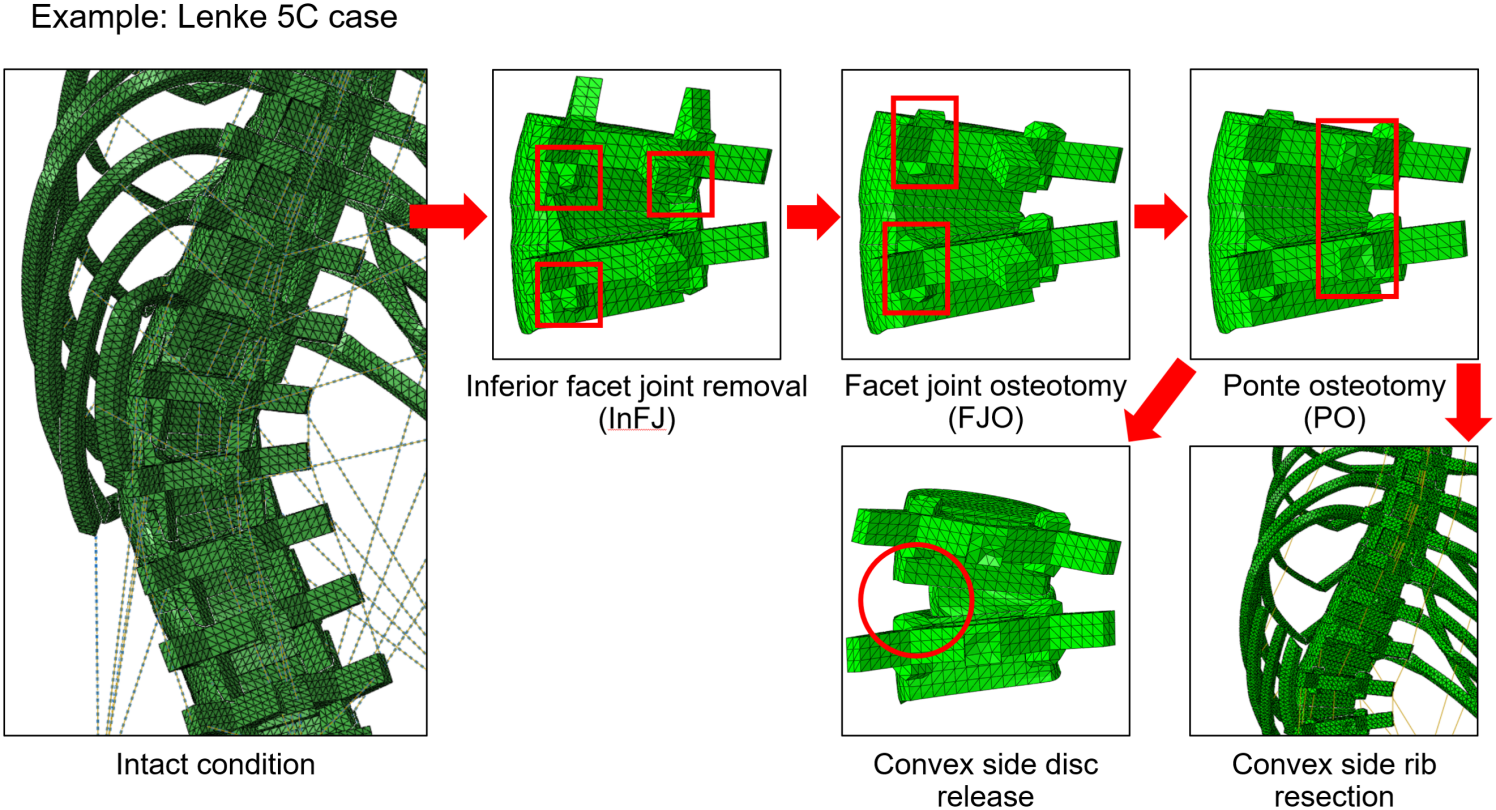
Surgical manoeuvres simulated in FE modelling to assess curve flexibility. A stepwise release was performed via mesh editing to analyse the curve flexibility change. The convex side disc release and rib resection were both conducted following the PO stage. The resected regions were marked in red.

Five surgical manoeuvres were analysed (Figure 5) following the sequential release procedure considered in clinical practice [16]: inferior facet joint (InFJ) removal, total facet joint osteotomy (FJO), ponte osteotomy (PO) and two extensive spinal release techniques for severe scoliosis—convex side IVD release and convex side rib resection. For convex side IVD release, IVDs at the convex side of the apical vertebrae were exposed and the lateral quarter of the annulus fibrosus was excised [30]. Both extensive release manoeuvres were performed following the PO stage.

## 3 Results

### 3.1 Generated Scoliosis Models

**FIGURE 6:**
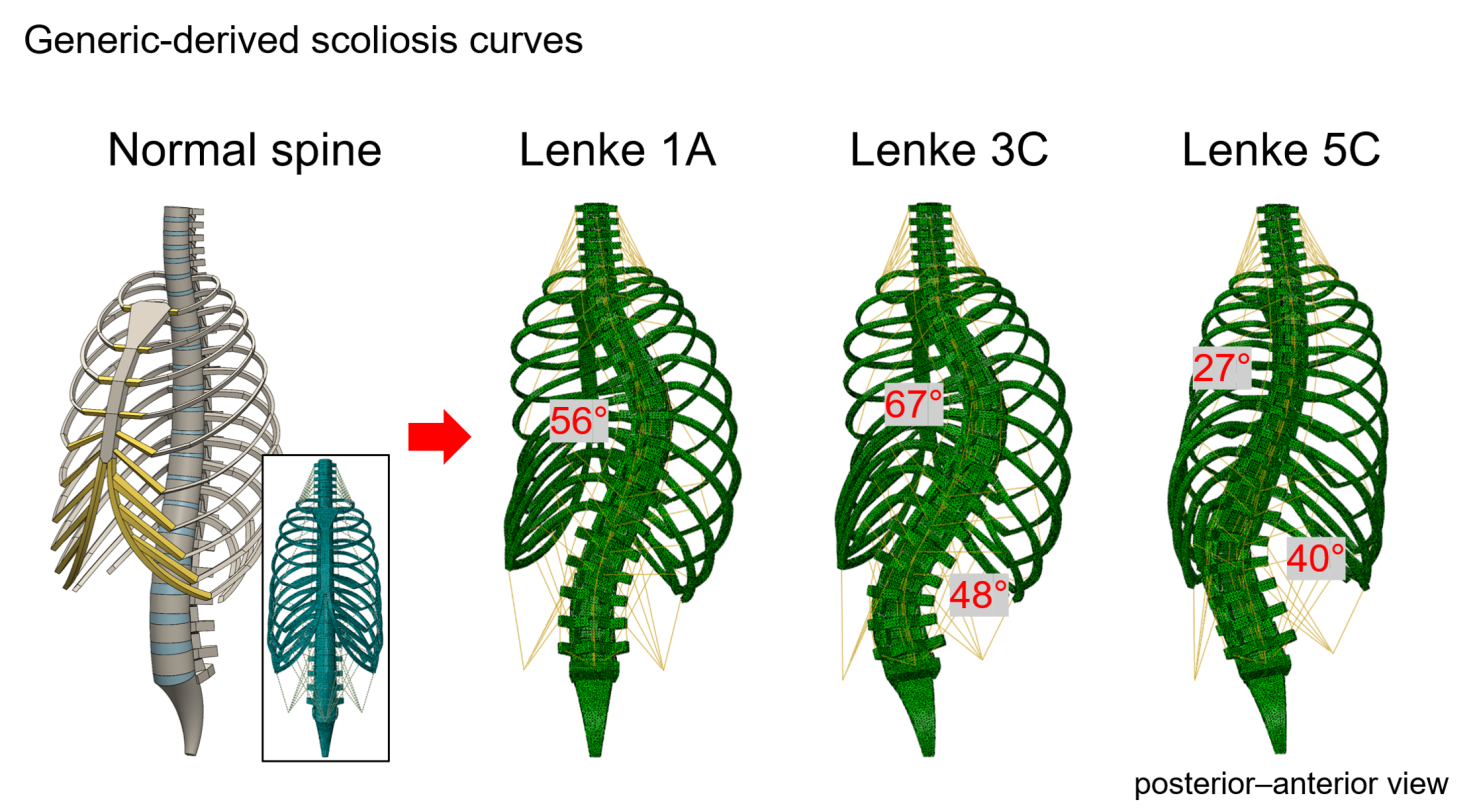
Generic‐derived scoliosis curves for curve flexibility assessment.

**FIGURE 7:**
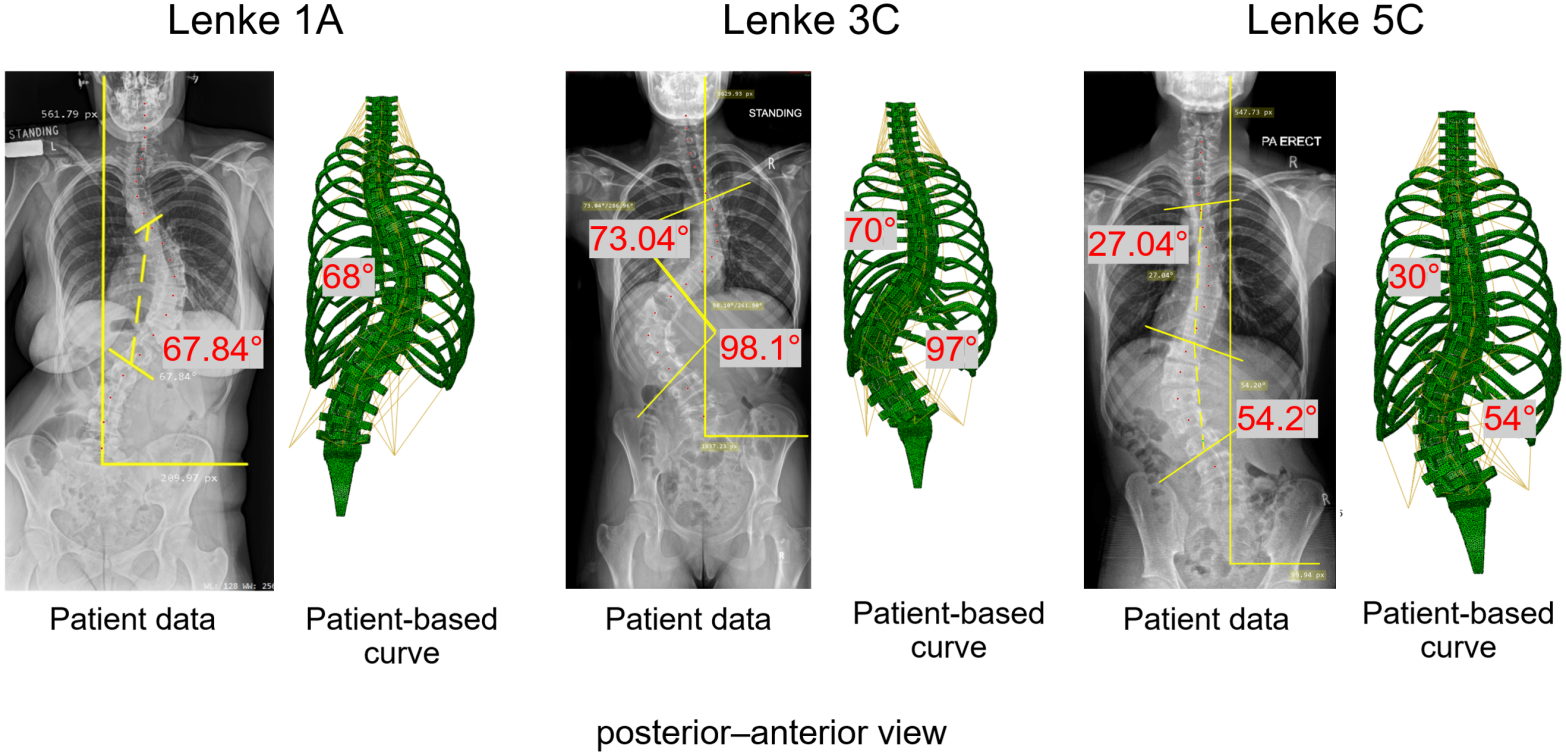
Generated patient‐based scoliosis curves for traction force analysis. The figures were scaled for better demonstration.

Using the displacement‐based shaping approach and by modifying the materials of spine components, we developed three generic‐derived scoliosis models (Figure 6) specifically for flexibility assessments and three patient‐based scoliosis curves (Figure 7) for the traction force estimation, representing Lenke 1A, 3C and 5C cases. Three generic‐derived scoliosis models were all set to the same spinal height for the flexibility tests to minimise errors arising from inconsistent model dimensions. Specifically, the models represented: a 56° major thoracic curve (1A); a double‐major curve including a 67° thoracic curve and a 48° lumbar curve (3C); and a 40° thoracolumbar with a 27° secondary curve (5C).

Three patient‐based curves represented severe cases with the same Lenke classification as the generic‐derived curves. However, a uniform spinal height was not maintained due to patient‐specific distributions of vertebral displacements along the spine. Three cases were: a 68° major thoracic curve (1A); a double‐major curve including a 70° thoracic curve and a 97° lumbar curve (3C); and a 54° thoracolumbar with a 30° secondary curve (5C). These curves closely followed the corresponding patient spines and were compared against the patients' radiographs. The mean difference between the patients' Cobb angles and those of the generated curves was 1.41°, which was below the commonly accepted 5° uncertainty in Cobb angle measurements; therefore, it was not considered clinically significant [29].

To evaluate whether the updated nodal coordinates introduced substantial element distortion, mesh quality was assessed after the scoliotic shaping procedure using generic‐derived scoliotic models (Lenke 1A, 3C and 5C). The baseline generic model and the generated AIS models were compared using the tetrahedral element quality criteria defined in this study, including minimum face angle (≥ 10°), maximum face angle (≤ 160°), aspect ratio (≤ 5) and shape factor (≥ 0.0001) [31]. Only negligible differences were observed in the proportions of elements satisfying the defined thresholds, indicating that the overall mesh quality was well preserved following the shaping procedure. Detailed comparisons are provided in Table S2.

### 3.2 Traction Force Prediction in STR Approach

The clinical records from three patients who were diagnosed using the STR approach and the curve flexibilities predicted for the corresponding patient‐based curves are summarised in Table 1.

**TABLE 1:** Comparison of curve flexibilities between clinical cases and patient‐based curves under STR and the required traction forces.

Lenke type	Preoperativeclinical diagnosis^a^						Simulation using patient‐based curves							
	Before traction		Aftertraction		Curveflexibility		Beforetraction		Traction region	After traction		Curveflexibility		Tractionforce
	(°)		(°)		(%)		(°)			(°)		(%)		(N)
1A	68		40		41.2		67.7		Cervical	41		39.4		376.6
									Axillae	36.3		46.4		516
3C	73	98	57	62	21.9	36.7	70.3	96.6	Cervical	39.7	79.4	43.5	17.8	414.5
										119.1		28.7		
	171		119		30.4		167		Axillae	52.0	61.5	26	36.3	1349
										113.6		32		
5C	27	54	20	25	25.9	53.7	29.5	53.8	Cervical	4.6	38.5	84.1	28.7	760
										43.1		48.1		
	81		45		44		83		Axillae	15.3	35.4	47.3	34.5	1092
										50.7		39		

*Note:* The major curves are in dark blue; the total Cobb angles recorded are in light blue. Tractions applied at the cervical region and axillae are in light grey and dark grey, respectively. Cervical and axillary traction represent two independent STR loading conditions and the corresponding traction forces were not applied simultaneously.

^a^ Clinical records from three patients at the time of preoperative diagnosis.

From the perspective of the total Cobb angle change (marked in light blue), the overall spinal flexibility was well reproduced. The mean difference in Cobb angle reduction between the clinical records and the FE predictions was 2.96°. In terms of the calculated curve flexibility, the mean difference between the clinical records and the FE results was 2.53% when traction was applied at the cervical region and was 3.93% when traction was applied at the axillae. When focusing on the local curve correction, the mean difference in curve flexibility was 11.3% for axillary traction and 30.9% for cervical traction.

For all simulations for reproducing the clinical recorded curve flexibility, the smallest traction force occurred in Lenke 1A case with a cervical traction of 376.6 N (≈ 38.4 kg). For severe cases, the required traction forces at the axillae exceeded 100 kg, with the largest recorded force reaching 1349 N (≈ 137.5 kg) for a nearly 100° thoracolumbar curve.

### 3.3 Curve Flexibility Assessments

Differences in curve flexibility were interpreted using the previously adopted 5° Cobb angle uncertainty threshold [29]. Changes below this threshold were not considered clinically significant and were therefore considered as minor improvements in curve flexibility.

**FIGURE 8:**
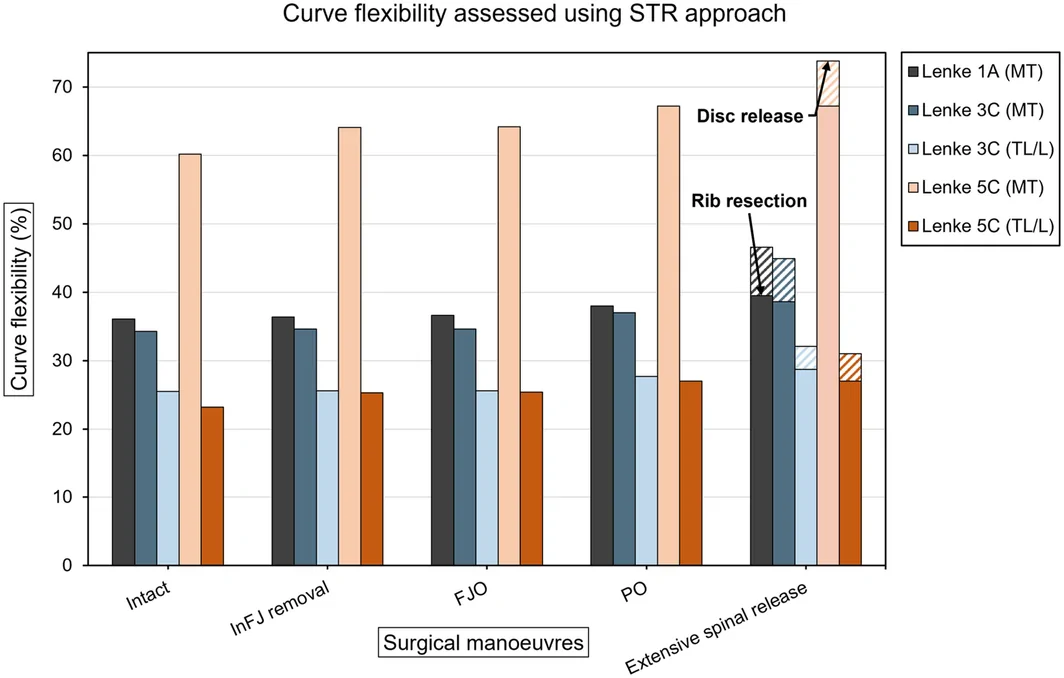
Curve flexibility assessed using STR approach across different surgical manoeuvres. Both the major thoracic (MT) and thoracolumbar/lumbar (TL/L) curve changes were presented. For extensive spinal release techniques, the lower bounds represent the curve flexibility achieved with the rib resection manoeuvre, and the upper bounds (shaded bars) represent the corresponding results for disc release.

**FIGURE 9:**
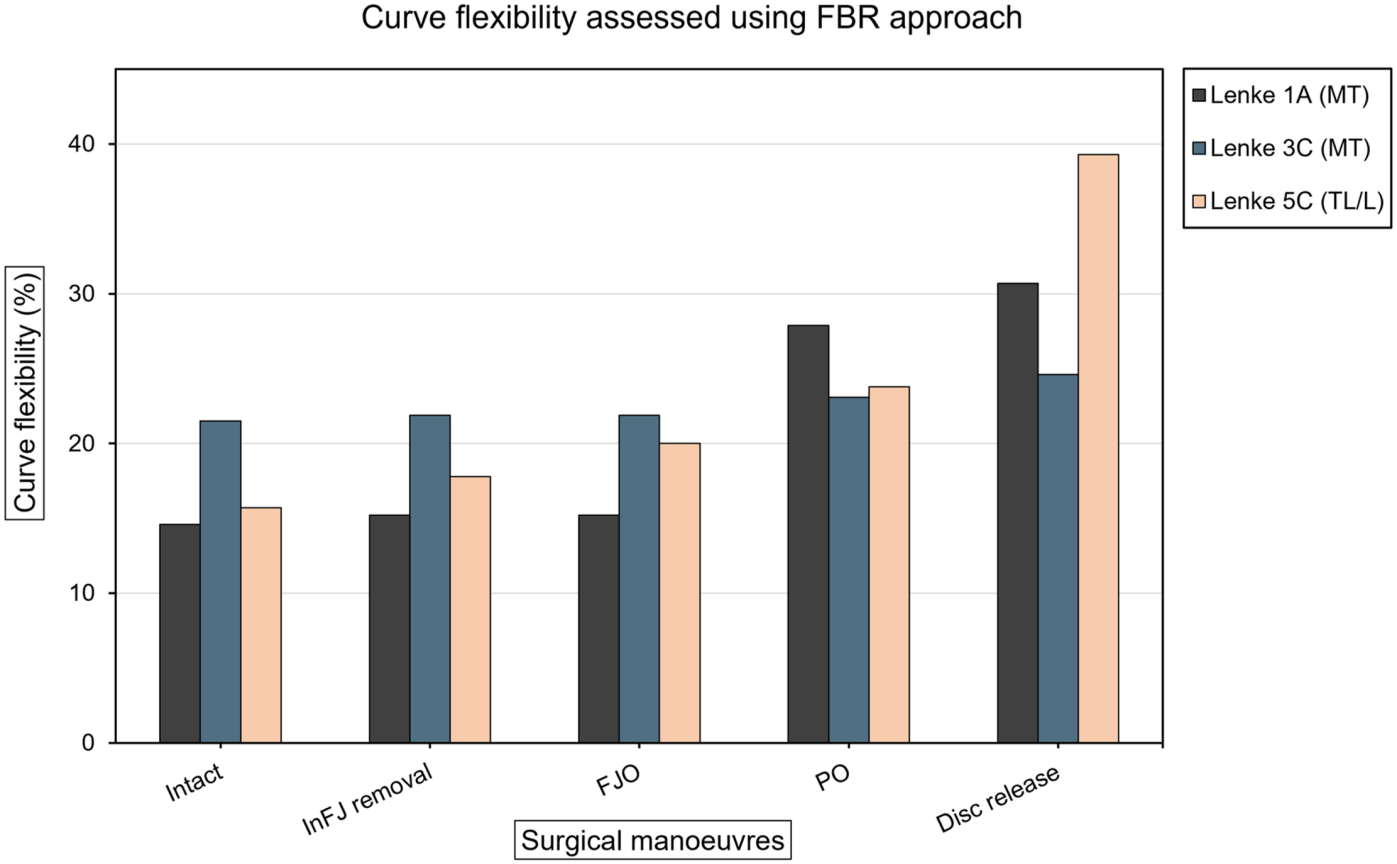
Curves flexibility assessed using FBR approach across different surgical manoeuvres.

Based on the traction force prediction, the minimum predicted traction force (376 N) was applied at the cervical region for the curve flexibility assessments of the three generic‐derived scoliosis curves. In all cases, the effect of different surgical manoeuvres was examined. Both the major thoracic (MT) and thoracolumbar/lumbar (TL/L) curve changes were measured and are presented for the STR approach (Figure 8, Table S3), while for the FBR approach, only the major curves were recorded and the rib resection manoeuvre was not performed (Figure 9, Table S4).

Under the STR approach, the curve flexibility increased with stepwise posterior releases, while the two extensive releases produced the largest changes (Figure 8). For Lenke 1A, the curve flexibility increased steadily from intact condition to PO (36.1%–38%) and increased the most after disc release (46.6%), with rib resection only resulting in 39.5%. For Lenke 3C, both thoracic and lumbar curves exhibited gradual increases from InFJ removal to PO, followed by a larger gain after disc release (MT 44.9%, TL/L 32.1%), while the rib resection resulted in smaller improvements (MT 38.6%, TL/L 28.7%). Lenke 5C showed high curve flexibility for the thoracic curve from intact condition to PO (60.2%–67.2%) and reached peak after disc release (73.8%), while the lumbar curve flexibility increased modestly (23.2%–31%). The rib resection manoeuvre did not improve the curve flexibility for Lenke 5C type.

Under the FBR approach, the curve flexibility varied differently across three Lenke types (Figure 9). Lenke 1A increased from low flexibility (14.6%) at intact condition to 27.9% after PO and reached 30.7% after disc release. Lenke 3C showed modest improvement in the thoracic curve from 21.5% to 24.6% throughout the spinal release procedures. Lenke 5C exhibited the greatest improvement in curve flexibility, from 15.7% to 39.3%.

## 4 Discussion

This study presented a procedure that can generate clinically representative AIS geometries from a well‐assessed generic adolescent full‐spine FE model [21]. By applying displacements on vertebrae and modifying the relative stiffness of spine components, the generated scoliosis models satisfactorily replicated morphological characteristics of the scoliotic spines, including wedge‐shaped vertebral and IVD geometries, vertebral axial rotations and ribcage deformity. The resulting scoliotic mesh coordinates were then used to replace the original nodal coordinates of the normal spine model to form FE scoliosis models, retaining the full ligament and muscle components for biomechanical assessments. In this study, two radiographic approaches (STR and FBR) were performed to analyse the curve flexibility change due to five types of surgical manoeuvres. Under STR, a practical procedure was presented to help estimate the required traction force (376 N). Unlike patient‐specific modelling, which requires FE model construction from full 3D scans of the patient, the proposed approach provides fast approximate answers to questions related to flexibility for different scoliotic patterns.

The proposed procedure for generating scoliotic spines from a baseline normal spine model is conceptually similar to the mesh‐morphing technique [31], which computationally deforms meshes to target scoliotic geometries. However, this approach requires patient‐specific data as a reference to define the target shape and is therefore less suitable for the generation of broadly applicable scoliosis models. At present, such generic scoliosis models remain scarce. Our proposed modelling procedure is novel and straightforward, enabling the development of a wide range of scoliosis curve types by editing the input file of the generic normal spine model.

A key advantage of developing generic scoliosis models is that all generated spines inherit the same baseline material definitions and overall dimensions. This improves comparability across simulations by reducing uncertainties due to inconsistent material properties or scale differences, thereby enabling more direct comparison of flexibility outcomes.

For the traction force estimation, the literature reported findings that were broadly consistent with those presented in this study. In experiments on idiopathic scoliosis patients (mean age 17.8 years) [26], achieving a mean curve flexibility of 32.5% required an average cervical traction force of 32.5 kg, which was approximately 53% of the tested patient's body weight and the traction was applied up to the maximum patient tolerance. Our results presented a slightly higher flexibility of 39.4%, requiring cervical traction of 38.4 kg. In a separate study on AIS patients [27], the mean age of patients was 15.1 years, which closely matched the age represented by the generic normal spine model used in this study [21]. Achieving a mean curve flexibility of 26.3% required an average axillary traction force of 81.5 kg, which was approximately 1.5 times the tested patient's body weight. For severe cases, such as a 70° thoracolumbar curve, the recorded traction force reached 120 kg [27]. In our results, the larger traction forces were all observed for axillary traction on thoracolumbar curves (Lenke 3C and 5C cases), with the largest recorded force reaching 137.5 kg for a 98° thoracolumbar curve. Higher traction demands in thoracolumbar curves reflected the resistance from the lumbopelvic musculature (iliopsoas were assigned the highest stiffness among the modelled muscles in the generic normal spine model [21]), which contributes to lumbar stability [32]. Although these findings are not a strong validation of traction force prediction as the applied traction magnitude is highly dependent on the individual patient, they provide a practical indication of the traction range required to elicit clinically meaningful flexibility using our generated scoliosis models.

For curve flexibility assessments, this study did not aim to draw a direct comparison between STR and FBR approaches. A key reason was the uncertainty in the traction force applied for the STR simulations, which was unable to ensure that the STR and FBR flexibility results represent matched measurements from the same patient. In addition, the FBR outcomes are sensitive to the fulcrum position, particularly for double major curves, further limiting the validity of any comparison.

Despite these constraints, distinct responses were observed across tested curve types and surgical manoeuvres. Under STR, flexibility variations of different scoliosis patterns were relatively consistent, showing a more regular and progressive increase along the stepwise spinal release. In particular, for both the major and secondary curves, facetectomy produced limited improvements in curve flexibility. This finding aligned with a previous in vitro study showing that facetectomy alone did not substantially improve flexibility in lateral bending [16], which was consistent with the STR approach as it similarly probes curve flexibility in the coronal plane. In contrast, FBR appeared more sensitive to the curve region, and it demonstrated more effective correction in lumbar curves. Similar to STR results, facetectomy did not provide additional improvements in flexibility in most scoliosis patterns, except for the major lumbar curve. This is because the correction at the lumbar region was less constrained by the thoracic cage, and the lumbar spine generally has a larger range of motion than the thoracic spine [21, 33]. The PO conduct provided a significantly larger increase in flexibility in Lenke 1A than in Lenke 3C. This may reflect that FBR imposed a localised bending moment around the fulcrum, and in a double major curve case, the correction at one curve can be constrained by the coupled stiffness of the second structural curve, thereby reducing the net correction. Among the investigated surgical manoeuvres, lateral IVD release provided the most pronounced increases in flexibility, especially for Lenke 5C. This is because the IVD is a primary contributor to spine stability [34] and transmits compressive loads between vertebrae. Thus, releasing IVDs directly reduces resistance to lateral deformation, especially when releasing lumbar IVDs where there is no ribcage constraint.

There is currently no consensus on flexibility thresholds that should trigger particular release or osteotomy selections across different scoliosis patterns. Only a preliminary study [35] analysed the flexibility thresholds for the FJO and PO conducts based on the historical patients treated in hospital. Our study provided a valuable perspective on the optimal flexibility for different types of curves and spinal release techniques. Future work on determining the ideal flexibility thresholds will incorporate the correction outcomes to establish a systematic investigation.

This study is subject to several limitations. First, the scoliotic geometry developed in this study was implemented as an updated mesh configuration by nodal coordinate replacement. This implies a loss of detailed geometric information that would be required for implant‐related modifications, such as accurate pedicle screw trajectory planning, drilling or further vertebral modifications. In such cases, reconstruction of vertebral geometry from orphan meshes would be necessary. Nevertheless, implant placement and bone modifications were not the focus of this study. Second, although the scoliotic shaping procedure generated asymmetrical changes in the pedicles, laminae and other posterior vertebral components together with vertebral rotation, wedging and ribcage deformation, these local morphological features were not individually matched to the exact anatomy for patient‐specific cases. Scoliosis involves not only changes in global spinal curvature but also substantial regional and intra‐vertebral morphological changes [31, 36]. Therefore, current patient‐based morphing that reproduces only the overall spinal curvature while neglecting local scoliotic features may reduce anatomical fidelity and potentially affect local biomechanical predictions. This is particularly relevant for analyses focusing on local stress distributions (e.g., IVD compressive stresses [37]), posterior vertebral biomechanics or implant–bone interaction. However, the patient‐based models developed in this study were intended as rapid approximations of patient spinal morphology rather than as high‐fidelity patient‐specific simulations. Likewise, ribcage deformation was not reproduced with high biofidelity, particularly for patient‐based models when comparing the resulting patient‐based curves with the corresponding patient radiographs, where the overall thoracic cage morphology did not match precisely. This highlights the need for further investigation into ribcage constraint definitions and thoracic coupling during the spine‐deforming procedure. Future studies could incorporate three‐dimensional reconstruction techniques [38, 39] to better characterise changes in rib morphology related to scoliosis, thereby enabling a more accurate reproduction of the deformed ribcage contour. Third, the muscular representation was simplified to account only for passive effects, assuming that patients remained fully relaxed during the flexibility tests. This neglects potential voluntary muscle activation in response to pain or discomfort, which may provide additional resistance during tests and consequently influence the predicted traction force required for STR. Nevertheless, the predicted traction forces remained within the approximate range reported in the limited available clinical data [26, 27]. Fourth, the material properties in this study remained representative of a healthy spine due to the limited availability of AIS literature quantifying disease‐related material variations relative to normal spines. This simplification may have contributed to the higher traction forces predicted in severe cases, particularly for thoracolumbar curves presented in this study. In addition, the adopted material properties in the present model were largely obtained from experimental studies that were not specifically conducted on adolescent populations. Age‐related differences in spinal tissue properties may therefore affect the predicted mechanical response, particularly the magnitude of stiffness‐dependent outcomes such as range of motion, internal stresses and the external forces required to achieve a given correction. Incorporating age‐specific or appropriately scaled material properties [37] would improve the physiological representation of the present 15‐year‐old model. Regarding the flexibility tests, similar to other radiographic approaches, STR and FBR primarily quantify the coronal correction and cannot capture changes in apical vertebral rotation during preoperative diagnosis [40].

By matching scoliosis curves with their clinically evaluated flexibility, the developed models can help surgeons decide which surgical manoeuvres are required for correction. Of particular interest to the authors is the assessment of different combinations of surgical conducts across varying curve flexibilities. The generic‐derived scoliosis models can be used to compare AIS corrections on the same scoliosis type, as well as to compare the performance of one surgical conduct across different scoliosis cases. Ultimately, this may support the development of a reference guide to help spinal surgeons evaluate the merits and feasibility of different treatment approaches.

## Funding

The authors have nothing to report.

## Ethics Statement

The authors have nothing to report.

## Conflicts of Interest

The authors declare no conflicts of interest.

## Supporting information


**Table S1:** Summary of material properties used for the generic adolescent spine model [21].
**Table S2:** Mesh quality assessment comparing generated AIS models with baseline generic model.
**Table S3:** Curve flexibility assessed using STR method across different surgical manoeuvres.
**Table S4:** Curve flexibility assessed using FBR method across different surgical manoeuvres.

## Data Availability

Most data derived from public domain sources. Data for patient‐based models is restricted due to ethical concerns.

## References

[1] S. L. Weinstein , L. A. Dolan , J. C. Y. Cheng , A. Danielsson , and J. A. Morcuende , “Adolescent Idiopathic Scoliosis,” Lancet 371, no. 9623 (2008): 1527–1537, 10.1016/S0140-6736(08)60658-3. 18456103

[2] J. A. Janicki and B. Alman , “Scoliosis: Review of Diagnosis and Treatment,” Paediatrics & Child Health 12, no. 9 (2007): 771–776, 10.1093/pch/12.9.771. 19030463 PMC2532872

[3] C. He and M.‐S. Wong , “Spinal Flexibility Assessment on the Patients With Adolescent Idiopathic Scoliosis: A Literature Review,” Spine 43, no. 4 (2018): E250–E258, 10.1097/BRS.0000000000002276. 28604491

[4] M. Khodaei , C. Pacheco‐Pereira , S. Trac , A. Chan , L. H. Le , and E. Lou , “Radiographic Methods to Estimate Surgical Outcomes Based on Spinal Flexibility Assessment in Patients Who Have Adolescent Idiopathic Scoliosis: A Systematic Review,” Spine Journal 18, no. 11 (2018): 2128–2139, 10.1016/j.spinee.2018.06.344. 29959103

[5] M. B. Janjua , J. C. Tishelman , D. Vasquez‐Montes , et al., “The Value of Sitting Radiographs: Analysis of Spine Flexibility and Its Utility in Preoperative Planning for Adult Spinal Deformity Surgery,” Journal of Neurosurgery: Spine 29, no. 4 (2018): 414–421, 10.3171/2018.2.SPINE17749. 29979136

[6] J. P. Little and C. J. Adam , “The Effect of Soft Tissue Properties on Spinal Flexibility in Scoliosis: Biomechanical Simulation of Fulcrum Bending,” Spine 34, no. 2 (2009): E76–E82, 10.1097/BRS.0b013e31818ad584. 19139657

[7] J. Li , S. Hwang , F. Wang , et al., “An Innovative Fulcrum‐Bending Radiographical Technique to Assess Curve Flexibility in Patients With Adolescent Idiopathic Scoliosis,” Spine 38, no. 24 (2013): E1527–E1532, 10.1097/BRS.0b013e3182a58e89. 24220308

[8] K. Cheung and K. Luk , “Prediction of Correction of Scoliosis With Use of the Fulcrum Bending Radiograph,” Journal of Bone and Joint Surgery. American Volume 79, no. 8 (1997): 1144–1150. 9278073 10.2106/00004623-199708000-00005

[9] V. Y. T. Hui , S. T. Y. Cheung , J. P. Y. Cheung , and P. W. H. Cheung , “How Accurate Are Fulcrum Bending Radiographs in Estimating Postoperative Outcomes in Adolescent Idiopathic Scoliosis? A Systematic Review and Meta‐Analysis,” Clinical Orthopaedics and Related Research 483, no. 10 (2025): 1951–1965, 10.1097/CORR.0000000000003468. 40132870 PMC12453376

[10] R. W. Liu , A. L. Teng , D. G. Armstrong , C. Poe‐Kochert , J. P. Son‐Hing , and G. H. Thompson , “Comparison of Supine Bending, Push‐Prone, and Traction Under General Anesthesia Radiographs in Predicting Curve Flexibility and Postoperative Correction in Adolescent Idiopathic Scoliosis,” Spine 35, no. 4 (2010): 416–422, 10.1097/BRS.0b013e3181b3564a. 20110844

[11] K. Siribumrungwong and N. Dhanachanvisith , “A Comparative Study of Supine Traction, Supine Side‐Bending Radiographs, and Supine MRI to Determine Coronal Flexibility in Degenerative Lumbar Scoliosis Patients,” Spine Deformity 11, no. 2 (2023): 423–432, 10.1007/s43390-022-00615-4. 36402926

[12] G. A. La Maida , E. Gallazzi , F. Ramella , et al., “What Is the Role of Traction Test Radiographs in the Preoperative Planning of Adolescent Idiopathic Scoliosis?,” Journal of Clinical Medicine 12, no. 22 (2023): 6986, 10.3390/jcm12226986. 38002604 PMC10671893

[13] F. Galbusera , M. Van Rijsbergen , K. Ito , J. M. Huyghe , M. Brayda‐Bruno , and H.‐J. Wilke , “Ageing and Degenerative Changes of the Intervertebral Disc and Their Impact on Spinal Flexibility,” European Spine Journal 23, no. Suppl 3 (2014): 324–332, 10.1007/s00586-014-3203-4. 24482074

[14] A. Fujiwara , T.‐H. Lim , H. S. An , et al., “The Effect of Disc Degeneration and Facet Joint Osteoarthritis on the Segmental Flexibility of the Lumbar Spine,” Spine 25, no. 23 (2000): 3036–3044. 11145815 10.1097/00007632-200012010-00011

[15] J. H. Yang , A. W. Bhandarkar , H. N. Modi , et al., “Short Apical Rib Resections Thoracoplasty Compared to Conventional Thoracoplasty in Adolescent Idiopathic Scoliosis Surgery,” European Spine Journal 23, no. 12 (2014): 2680–2688, 10.1007/s00586-014-3299-6. 24719039

[16] R. M. Holewijn , T. P. Schlösser , A. Bisschop , et al., “How Does Spinal Release and Ponte Osteotomy Improve Spinal Flexibility? The Law of Diminishing Returns,” Spine Deformity 3, no. 5 (2015): 489–495, 10.1016/j.jspd.2015.03.006. 27927536

[17] C. Ottardi , F. Galbusera , A. Luca , et al., “Finite Element Analysis of the Lumbar Destabilization Following Pedicle Subtraction Osteotomy,” Medical Engineering & Physics 38, no. 5 (2016): 506–509, 10.1016/j.medengphy.2016.02.002. 26968784

[18] N. Shekouhi , S. Tripathi , A. Theologis , et al., “Evaluating the Biomechanical Effects of Pedicle Subtraction Osteotomy at Different Lumbar Levels: A Finite Element Investigation,” Spine Journal 24, no. 11 (2024): 2191–2203, 10.1016/j.spinee.2024.07.005. 39097103

[19] C. R. D'Andrea , A. F. Samdani , and S. Balasubramanian , “Patient‐Specific Finite Element Modeling of Scoliotic Curve Progression Using Region‐Specific Stress‐Modulated Vertebral Growth,” Spine Deformity 11, no. 3 (2023): 525–534, 10.1007/s43390-022-00636-z. 36593421 PMC10147794

[20] M. Gay , N. Cobetto , C. Caouette , et al., “Patient‐Specific Biomechanical Modeling of Intraoperative Scoliosis Correction on the 2‐Year Outcomes for Thoracolumbar/Lumbar Vertebral Body Tethering,” Spine Deformity 14, no. 1 (2026): 31–38, 10.1007/s43390-025-01166-0. 40880062

[21] Y. Su , A. I. Tsirikos , V. Koutsos , and P. Pankaj , “Development and Assessment of a Novel Generic Finite Element Spine Model for Clinical Applications,” International Journal for Numerical Methods in Biomedical Engineering 41, no. 9 (2025): e70098, 10.1002/cnm.70098. 40981663 PMC12452808

[22] P. L. Mente , D. D. Aronsson , I. A. Stokes , and J. C. Iatridis , “Mechanical Modulation of Growth for the Correction of Vertebral Wedge Deformities,” Journal of Orthopaedic Research 17, no. 4 (1999): 518–524, 10.1002/jor.1100170409. 10459757

[23] S.‐A. Scherrer , M. Begon , A. Leardini , C. Coillard , C.‐H. Rivard , and P. Allard , “Three‐Dimensional Vertebral Wedging in Mild and Moderate Adolescent Idiopathic Scoliosis,” PLoS One 8, no. 8 (2013): e71504, 10.1371/journal.pone.0071504. 23977058 PMC3744570

[24] J. Clin , C.‐É. Aubin , N. Lalonde , S. Parent , and H. Labelle , “A New Method to Include the Gravitational Forces in a Finite Element Model of the Scoliotic Spine,” Medical & Biological Engineering & Computing 49, no. 8 (2011): 967–977, 10.1007/s11517-011-0793-4. 21728065

[25] C. Murata , D. Matsuoka , T. Murase , and J. Takahashi , “Proteinuria From First‐Morning Urine in a Child due to Brace Treatment for Adolescent Idiopathic Scoliosis,” CEN Case Reports 11, no. 4 (2022): 490–493, 10.1007/s13730-022-00708-z. 35532856 PMC9626715

[26] N. Te Hennepe , M. Spruit , M. H. Pouw , M. Hinderks , and P. Heesterbeek , “Supine Traction Versus Prone Bending Radiographs for Assessing the Curve Flexibility in Spinal Deformity,” Global Spine Journal 12, no. 7 (2022): 1345–1351, 10.1177/2192568220979136. 33504207 PMC9393981

[27] C. J. O'Neill , S. A. Brennan , C. Quinn , D. Brabazon , and P. J. Kiely , “Standardized Traction Versus Side‐Bending Radiographs in Adolescent Idiopathic Scoliosis: A Preliminary Study,” Journal of Pediatric Orthopaedics. Part B 28, no. 1 (2019): 17–21, 10.1097/BPB.0000000000000550. 30252796

[28] M. Islán Marcos , E. Lechosa Urquijo , F. Blaya Haro , R. D'Amato , E. Soriano Heras , and J. A. Juanes , “Behavior Under Load of a Human Shoulder: Finite Element Simulation and Analysis,” Journal of Medical Systems 43, no. 5 (2019): 132, 10.1007/s10916-019-1248-y. 30941505

[29] C. Jin , S. Wang , G. Yang , E. Li , and Z. Liang , “A Review of the Methods on Cobb Angle Measurements for Spinal Curvature,” Sensors 22, no. 9 (2022): 3258, 10.3390/s22093258. 35590951 PMC9101880

[30] Z. Deng , P. Xiu , L. Wang , et al., “The Comparison of Posterior Intervertebral Release Combined With Posterior Column Osteotomy and Posterior Column Osteotomy Alone for the Treatment of Moderate‐To‐Severe Rigid Scoliosis: A Prospective Controlled Study,” Orthopaedic Surgery 16, no. 3 (2024): 594–603, 10.1111/os.13987. 38237925 PMC10925497

[31] P. Hadagali , J. R. Peters , and S. Balasubramanian , “Morphing the Feature‐Based Multi‐Blocks of Normative/Healthy Vertebral Geometries to Scoliosis Vertebral Geometries: Development of Personalized Finite Element Models,” Computer Methods in Biomechanics and Biomedical Engineering 21, no. 4 (2018): 297–324, 10.1080/10255842.2018.1448391. 29528253

[32] E. Andersson , L. Oddsson , H. Grundström , and A. Thorstensson , “The Role of the Psoas and Iliacus Muscles for Stability and Movement of the Lumbar Spine, Pelvis and Hip,” Scandinavian Journal of Medicine & Science in Sports 5, no. 1 (1995): 10–16, 10.1111/j.1600-0838.1995.tb00004.x. 7882121

[33] M. Dreischarf , T. Zander , A. Shirazi‐Adl , et al., “Comparison of Eight Published Static Finite Element Models of the Intact Lumbar Spine: Predictive Power of Models Improves When Combined Together,” Journal of Biomechanics 47, no. 8 (2014): 1757–1766, 10.1016/j.jbiomech.2014.04.002. 24767702

[34] P. R. Loughenbury , A. I. Tsirikos , and N. W. Gummerson , “Spinal Biomechanics–Biomechanical Considerations of Spinal Stability in the Context of Spinal Injury,” Orthopaedics and Traumatology 30, no. 5 (2016): 369–377, 10.1016/j.mporth.2016.07.010.

[35] Y. Liu , Z. Zhang , G. Zhu , and J. Liang , “Spinal Flexibility in Idiopathic Scoliosis: A Quantitative Approach to Grade I (Facet Joint Osteotomy, FJO) and Grade II (Ponte Osteotomy, PO) Osteotomy Techniques,” Journal of Orthopaedic Surgery 33, no. 2 (2025): 10225536251357770, 10.1177/10225536251357770. 40631396

[36] P. Hadagali , Subject‐Specific Finite Element Modeling of the Adolescent Thoracic Spine for Scoliosis Research (Drexel University, 2014).

[37] S. Balasubramanian , C. R. D'Andrea , G. Viraraghavan , and P. J. Cahill , “Development of a Finite Element Model of the Pediatric Thoracic and Lumbar Spine, Ribcage, and Pelvis With Orthotropic Region‐Specific Vertebral Growth,” Journal of Biomechanical Engineering 144, no. 10 (2022): 101007, 10.1115/1.4054410. 35466381

[38] D. Mitton , K. Zhao , S. Bertrand , et al., “3D Reconstruction of the Ribs From Lateral and Frontal X‐Rays in Comparison to 3D CT‐Scan Reconstruction,” Journal of Biomechanics 41, no. 3 (2008): 706–710, 10.1016/j.jbiomech.2007.09.034. 17981286

[39] S. Delorme , Y. Petit , J. A. de Guise , H. Labelle , C.‐E. Aubin , and J. Dansereau , “Assessment of the 3‐D Reconstruction and High‐Resolution Geometrical Modeling of the Human Skeletal Trunk From 2‐D Radiographic Images,” IEEE Transactions on Biomedical Engineering 50, no. 8 (2003): 989–998, 10.1109/TBME.2003.814525. 12892326

[40] T. Ibrahim , O. Gabbar , K. El‐Abed , M. Hutchinson , and I. Nelson , “The Value of Radiographs Obtained During Forced Traction Under General Anaesthesia in Predicting Flexibility in Idiopathic Scoliosis With Cobb Angles Exceeding 60,” Journal of Bone and Joint Surgery. British Volume 90, no. 11 (2008): 1473–1476, 10.1302/0301-620X.90B11.20690. 18978268

