# Supplementary material for: Evaluation of Curve Flexibility Using Scoliotic Spines Generated From a Generic Spine Model
Source: Int J Numer Method Biomed Eng. 2026 Sep 15;42(9):e70213. doi: 10.1002/cnm.70213 (PMC13578857; doi:10.1002/cnm.70213)
Supplement: Supplementary file 1 [file CNM-42-e70213-s001.docx]

| **Table S1. Summary of material properties used for the generic adolescent spine model [21].** | |  |
| --- | --- | --- |
| Spine components | Modulus of elasticity (MPa)/Poisson ratio | |
| Vertebral body | 3,000/0.3 | |
| Vertebral posterior | 12,000/0.3 | |
| Annulus fibrosus | 4.2/0.45 | |
| Nucleus pulposus | 1/0.49 | |
| Facet cartilage | 35/0.4 | |
| Costal cartilage | 10.5/0.2 | |
| Sternum | 12,000/0.3 | |
| Rib | 11,300/0.3 | |
| *Ligaments | Axial stiffness (N/mm) | |
| Anterior longitudinal ligament **_C, T, L_** | 16.00, 81.54, 8.74 | |
| Posterior longitudinal ligament **_C, T, L_** | 25.40, 29.09, 5.83 | |
| Ligamentum flavum **_C, T, L_** | 25.00, 68.76, 15.38 | |
| Interspinous ligament **_C, T, L_** | 7.74, 23.20, 0.19 | |
| Supraspinous ligament **_C, T, L_** | 7.74, 31.36, 2.39 | |
| Cartilage ligament **_C, T, L_** | 33.60, 1.98, 7.88 | |
| Intertransverse ligament **_C, T, L_** | 1.62, 4.80, 5.43 | |
| Musculature | Axial stiffness (N/mm) | |
| *Serratus posterior **_I, S_** | 0.068, 0.068 | |
| *Scalene **_A, M, P_** | 0.159, 0.124, 0.118 | |
| Iliopsoas | 113.44 | |
| Intercostals | 27.926 | |
| Combined components | 9.153 | |
| Rectus abdominis | 0.713 | |
| *The axial stiffness of ligaments is listed for cervical (C), thoracic (T) and lumbar (L) region respectively.  *The axial stiffness of serratus posterior is listed for inferior (I) and superior (S) components.  *The axial stiffness of scalene is listed for anterior, middle and posterior components. | |  |

| **Table S2. Mesh quality assessment comparing generated AIS models with baseline generic model** | | | |
| --- | --- | --- | --- |
| Baseline generic model |  |  |  |
| Criterion | Tet failed* | Failed % | Tet passed % |
| Min angle < 10° | 662 / 240,062 | 0.2758 | 99.7242 |
| Max angle > 160° | 60 / 240,062 | 0.0250 | 99.9750 |
| Aspect ratio > 5 | 598 / 240,062 | 0.2891 | 99.7109 |
| Shape factor < 0.0001 | 20 / 240,062 | 0.0083 | 99.9917 |
|  | | | |
| Lenke 1A model |  |  |  |
| Criterion | Tet failed | Failed % | Tet passed % |
| Min angle < 10° | 669 / 240,062 | 0.2787 | 99.7213 |
| Max angle > 160° | 56 / 240,062 | 0.0233 | 99.9767 |
| Aspect ratio > 5 | 675 / 240,062 | 0.2812 | 99.7188 |
| Shape factor < 0.0001 | 20 / 240,062 | 0.0083 | 99.9917 |
|  | | | |
| Lenke 3C model |  |  |  |
| Criterion | Tet failed | Failed % | Tet passed % |
| Min angle < 10° | 673 / 240,062 | 0.2803 | 99.7197 |
| Max angle > 160° | 60 / 240,062 | 0.0250 | 99.9750 |
| Aspect ratio > 5 | 673 / 240,062 | 0.2803 | 99.7197 |
| Shape factor < 0.0001 | 21 / 240,062 | 0.0088 | 99.9913 |
|  |  |  |  |
| Lenke 5C model |  |  |  |
| Criterion | Tet failed | Failed % | Tet passed % |
| Min angle < 10° | 666 / 240,062 | 0.2774 | 99.7226 |
| Max angle > 160° | 58 / 240,062 | 0.0242 | 99.9758 |
| Aspect ratio > 5 | 707 / 240,062 | 0.2945 | 99.7055 |
| Shape factor < 0.0001 | 19 / 240,062 | 0.0079 | 99.9921 |
| *The baseline generic model was meshed using 10-noded quadratic tetrahedral (Tet) elements (C3D10) and facet cartilage models were meshed using quadratic wedge elements (C3D15). Only the tetrahedral elements were assessed in this study, as facet cartilages were reconstructed using wedge elements in each generated AIS models. | | | |

| **Table S3. Curve flexibility assessed using STR method across different surgical manoeuvres** | | | | | | | | | | | | | | | |
| --- | --- | --- | --- | --- | --- | --- | --- | --- | --- | --- | --- | --- | --- | --- | --- |
| **STR** | | | | | | | | | | | | | | | |
| Lenke type | | Pre-op | | Intact | | InFJ | | FJO | | PO | | Disc release | | Rib resection | |
| 1A | Cobb angle (º) | 56.1 | | 35.9 | | 35.7 | | 35.6 | | 34.8 | | 30.0 | | 34.0 | |
|  | Curve flexibility (%) | - | | 36.1 | | 36.4 | | 36.6 | | 38.0 | | 46.6 | | 39.5 | |
| 3C | Cobb angle (º) | 67.0 | 47.7 | 44.0 | 35.6 | 43.8 | 35.5 | 43.8 | 35.5 | 42.2 | 34.5 | 36.9 | 32.4 | 41.1 | 34.1 |
|  | Curve flexibility (%) | - | - | 34.3 | 25.5 | 34.6 | 25.6 | 34.6 | 25.6 | 37.0 | 27.7 | 44.9 | 32.1 | 38.6 | 28.7 |
| 5C | Cobb angle (º) | 26.8 | 39.8 | 10.7 | 30.6 | 9.6 | 29.8 | 9.6 | 29.7 | 8.8 | 29.1 | 7.0 | 27.5 | 8.8 | 29.1 |
|  | Curve flexibility (%) | - | - | 60.2 | 23.2 | 64.1 | 25.3 | 64.2 | 25.4 | 67.2 | 27.0 | 73.8 | 31.0 | 67.2 | 27.0 |

| **Table S4. Curve flexibility assessed using FBR method across different surgical manoeuvres** | | | | | | | | | | | | | | |
| --- | --- | --- | --- | --- | --- | --- | --- | --- | --- | --- | --- | --- | --- | --- |
| **FBR** | | | | | | | | | | | | | | |
| Lenke type | | Pre-op | | Intact | | InFJ | | FJO | | PO | | Disc release | | Rib resection |
| 1A | Cobb angle (º) | 56.1 | | 47.9 | | 47.6 | | 47.6 | | 40.5 | | 38.9 | |  |
|  | Curve flexibility (%) | - | | 14.6 | | 15.2 | | 15.2 | | 27.9 | | 30.7 | |  |
| 3C | Cobb angle (º) | 67.0 | 47.7 | 52.6 | 47.9 | 52.3 | 48.0 | 52.3 | 48.1 | 51.5 | 48.3 | 50.5 | 49.0 |  |
|  | Curve flexibility (%) | - | - | 21.5 | -0.3 | 21.9 | -0.6 | 21.9 | -0.7 | 23.1 | -1.1 | 24.6 | -2.7 |  |
| 5C | Cobb angle (º) | 26.8 | 39.8 | 56.5 | 33.6 | 55.7 | 32.7 | 55.2 | 31.9 | 56.2 | 30.4 | 53.5 | 24.2 |  |
|  | Curve flexibility (%) | - | - | -110.6 | 15.7 | -107.7 | 17.8 | -105.9 | 20.0 | -109.6 | 23.8 | -99.8 | 39.3 |  |
